# IKK inhibition increases bortezomib effectiveness in ovarian cancer

**DOI:** 10.18632/oncotarget.4713

**Published:** 2015-07-20

**Authors:** Bipradeb Singha, Himavanth Reddy Gatla, Sai Phyo, Atish Patel, Zhe-Sheng Chen, Ivana Vancurova

**Affiliations:** ^1^ Department of Biological Sciences, St. John's University, Queens, NY 11439, USA; ^2^ Department of Pharmaceutical Sciences, St. John's University, Queens, NY 11439, USA

**Keywords:** bortezomib, IKK, interleukin-8, ovarian cancer, proteasome inhibition

## Abstract

Ovarian cancer is associated with increased expression of the pro-angiogenic chemokine interleukin-8 (IL-8, CXCL8), which induces tumor cell proliferation, angiogenesis, and metastasis. Even though bortezomib (BZ) has shown remarkable anti-tumor activity in hematological malignancies, it has been less effective in ovarian cancer; however, the mechanisms are not understood. We have recently shown that BZ unexpectedly induces the expression of IL-8 in ovarian cancer cells *in vitro*, by IκB kinase (IKK)-dependent mechanism. Here, we tested the hypothesis that IKK inhibition reduces the IL-8 production and increases BZ effectiveness in reducing ovarian tumor growth *in vivo*. Our results demonstrate that the combination of BZ and the IKK inhibitor Bay 117085 significantly reduces the growth of ovarian tumor xenografts in nude mice when compared to either drug alone. Mice treated with the BZ/Bay 117085 combination exhibit smallest tumors, and lowest levels of IL-8. Furthermore, the reduced tumor growth in the combination group is associated with decreased tumor levels of S536P-p65 NFκB and its decreased recruitment to IL-8 promoter in tumor tissues. These data provide the first *in vivo* evidence that combining BZ with IKK inhibitor is effective, and suggest that using IKK inhibitors may increase BZ effectiveness in ovarian cancer treatment.

## INTRODUCTION

Each year, ovarian cancer is diagnosed in nearly a quarter of a million women around the world, and is responsible for approximately 140, 000 deaths. Due to the asymptomatic nature of early disease, most women present with stage III and IV ovarian cancer, for which the standard of care remains surgery and platinum-based chemotherapy using cisplatin or carboplatin, and taxane agents [1, 2]. However, since most ovarian cancers relapse and become drug-resistant, the survival rates remain low [3–5]. The advanced stages of ovarian cancer are characterized by the increased expression of the pro-inflammatory and pro-angiogenic chemokine IL-8 (CXCL8), which induces tumor cell survival, proliferation, angiogenesis, and metastasis, and correlates with poor prognosis [6–12].

Even though proteasome inhibition by bortezomib (BZ; Velcade, PS-341) has shown remarkable anti-tumor activity in multiple myeloma, cutaneous T cell lymphoma, and other hematological malignancies, it has been less effective in solid tumors, including ovarian cancer [13–18]. However, the mechanisms are not fully understood. Nevertheless, BZ has been considered in combination with other therapies, especially the currently used cisplatin, since BZ prevents the cisplatin-induced degradation of cisplatin influx transporter, resulting in enhanced cisplatin uptake and tumor cell killing [19, 20]. Thus, understanding the mechanisms responsible for the resistance of ovarian carcinoma to BZ may lead to the development of more effective combination therapies.

The initial rationale behind BZ development and use was its inhibition of the inducible IκBα degradation by 26S proteasome, resulting in the inhibition of nuclear translocation of NFκB subunits and decreased expression of NFκB-dependent anti-apoptotic genes in multiple myeloma cells [21, 22]. However, studies from our laboratory have shown that proteasome inhibition also induces nuclear translocation of IκBα, which has a promoter specific effect on the suppression of NFκB-dependent genes [23, 24]. While most genes are inhibited by the nuclear IκBα, the IL-8 expression is IκBα independent [25]. Our recent *in vitro* studies have shown that the proteasome inhibition by BZ actually increases the IL-8 expression in cancer cells [26–28]. In ovarian cancer cells *in vitro*, the BZ increased IL-8 expression is mediated by IKKβ-dependent recruitment of S536-phosphorylated p65 to IL-8 promoter [27], suggesting that inhibition of IKK activity might increase BZ effectiveness in ovarian cancer.

In this study, we tested the hypothesis that IKK inhibition increases BZ effectiveness in reducing tumor growth *in vivo*, in ovarian cancer xenografts. We demonstrate that combination of BZ and the IKK inhibitor Bay 117085 significantly reduces ovarian tumor growth in nude mice when compared to either drug alone. Our results indicate that the underlying mechanisms involve decreased tumor levels of S536P-p65 and its decreased recruitment to IL-8 promoter in tumor tissue, resulting in reduced tumor mRNA levels and plasma concentrations of IL-8. These data suggest that combining BZ with IKK inhibitor is effective and may demonstrate a clinical benefit in the ovarian cancer treatment.

## RESULTS

### Suppression of BZ-induced IL-8 expression enhances BZ pro-apoptotic effect in ovarian cancer cells

**Figure 1:**
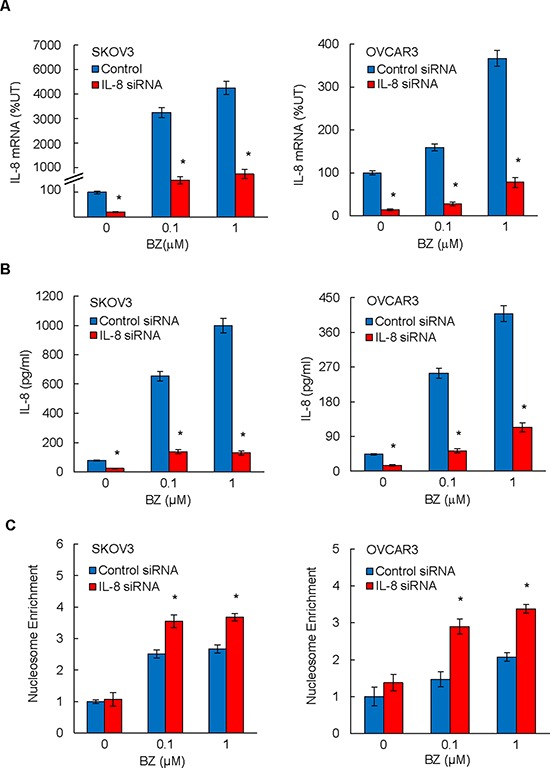
Suppression of BZ-induced IL-8 expression enhances BZ pro-apoptotic effect in ovarian cancer cells SKOV3 and OVCAR3 cells were transfected with control siRNA (blue columns) or IL-8 specific siRNA (red columns), treated 24 hours with 0, 0.1, and 1 μM BZ, and analyzed for IL-8 mRNA expression by real time RT-PCR **A.** and for IL-8 release by ELISA **B.** Apoptosis was analyzed by the cytoplasmic nucleosome enrichment assay in SKOV3 and OVCA3 cells transfected with control or IL-8 specific siRNA and incubated with 0, 0.1, and 1 μM BZ for 24 hours **C.** The values in Figures 1A–1C represent the mean +/–SE of four experiments. Asterisks denote a statistically significant (*p* < 0.05) change compared to cells transfected with the corresponding control siRNA.

Since we have recently shown that BZ increases the *in vitro* IL-8 expression in ovarian cancer cells [27], we wanted to determine whether the BZ-induced IL-8 expression is responsible for the decreased effectiveness of BZ in ovarian cancer cells. To address this hypothesis, we suppressed the IL-8 expression in BZ-treated ovarian cancer SKOV3 and OVCAR3 cells by siRNA, and evaluated apoptosis by cell death ELISA assay that quantifies the release of nucleosomes into the cytoplasm [28]. As expected, BZ considerably increased both IL-8 mRNA levels (Figure 1A) and IL-8 cytokine release (Figure 1B) in SKOV3 and OVCAR3 cells transfected with control siRNA. In cells transfected with IL-8 specific siRNA, the IL-8 expression and release were significantly decreased (Figures 1A and 1B).

BZ also increased apoptosis in SKOV3 and OVCAR3 cells transfected with control siRNA (Figure 1C); this is in an agreement with previous studies demonstrating that proteasome inhibition induces apoptosis in ovarian cancer cells [29, 30]. Importantly, the BZ-induced apoptosis was significantly increased in IL-8 siRNA transfected cells compared to cells transfected with control siRNA (Figure 1C), indicating that suppression of the BZ-induced IL-8 expression enhances the pro-apoptotic effect of BZ in ovarian cancer cells *in vitro*.

### IKK inhibitor Bay 117085 suppresses BZ induced IL-8 expression in ovarian cancer cells

**Figure 2:**
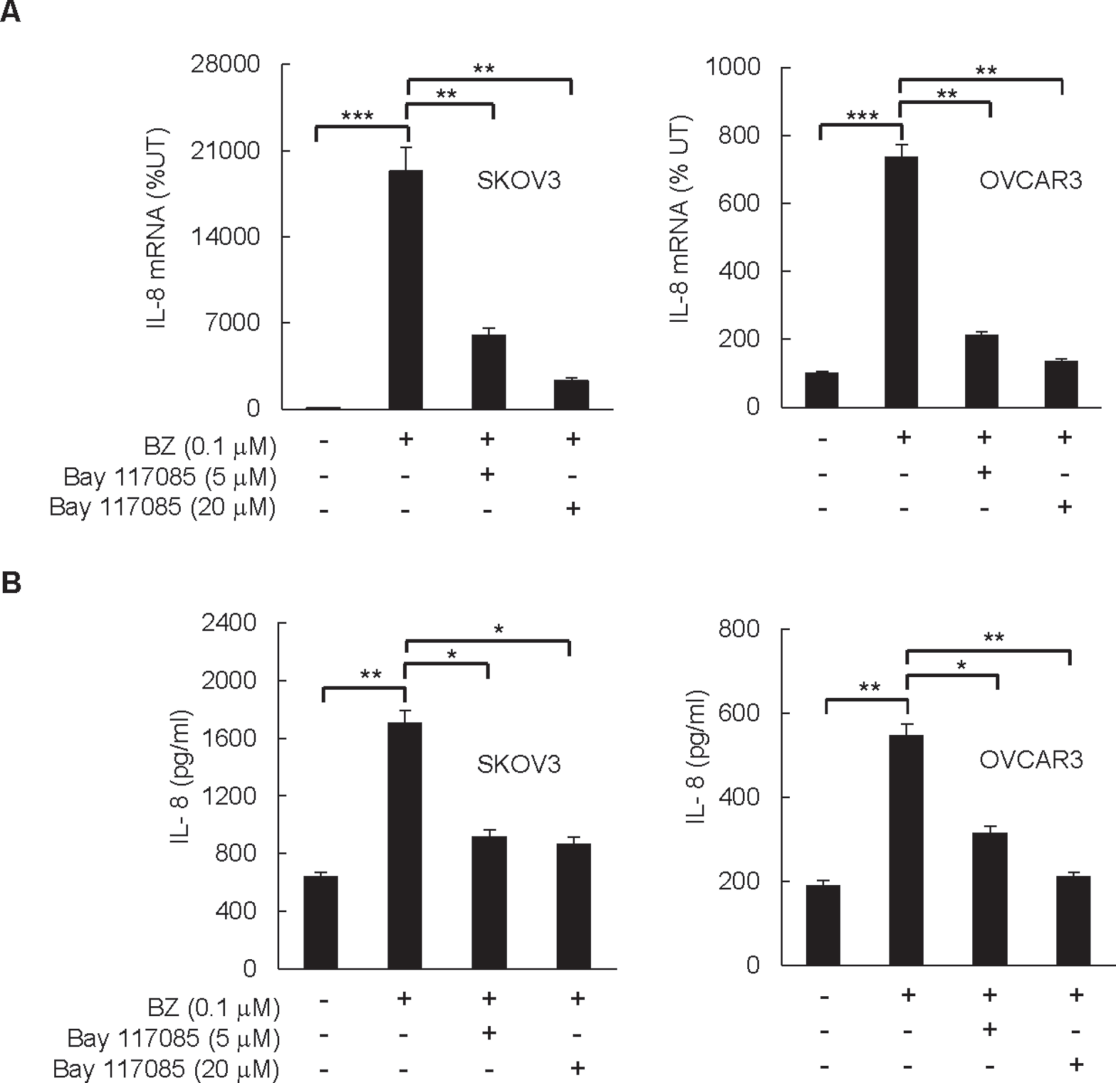
IKK inhibitor Bay 117085 suppresses BZ-induced IL-8 expression in ovarian cancer cells SKOV3 and OVCAR3 cells were pre-incubated 12 hours with 5 or 20 μM Bay 117085, treated 24 hours with 0.1 μM BZ, and analyzed for IL-8 mRNA expression by real time RT-PCR **A.** and for IL-8 release by ELISA **B.** The values in Figure 2 represent the mean +/–SE of four experiments. Asterisks denote a statistically significant change (*p < 0.05; **p < 0.01; ***p < 0.001).

Since our data demonstrated that suppression of IL-8 enhances the BZ pro-apoptotic effect (Figure 1), and since studies by Mabuchi *et al*. [31, 32] indicated that IKK inhibition by the soluble IKK inhibitor Bay 117085 induces apoptosis in ovarian cancer cells, we hypothesized that IKK inhibition by Bay 117085 inhibits the BZ-induced IL-8 expression in ovarian cancer cells. To test this hypothesis, SKOV3 and OVCAR3 cells were pre-incubated 12 hours with 0, 5, and 20 μM Bay 117085 [31], before 24-hour incubation with 0.1 μM BZ, which approximately corresponds to the clinically used BZ concentrations [33], and IL-8 mRNA levels and cytokine release were analyzed by RT-PCR and ELISA, respectively. As shown in Figure 2, Bay 117085 significantly decreased the BZ-induced IL-8 mRNA levels (Figure 2A) and cytokine release (Figure 2B) in ovarian cancer SKOV3 and OVCAR3 cells, suggesting that IKK inhibition by Bay 117085 may enhance the BZ effectiveness in ovarian cancer treatment.

### Combination of BZ and Bay 117085 enhances BZ effectiveness in reducing tumor growth in nude mice implanted with ovarian cancer xenografts

**Figure 3:**
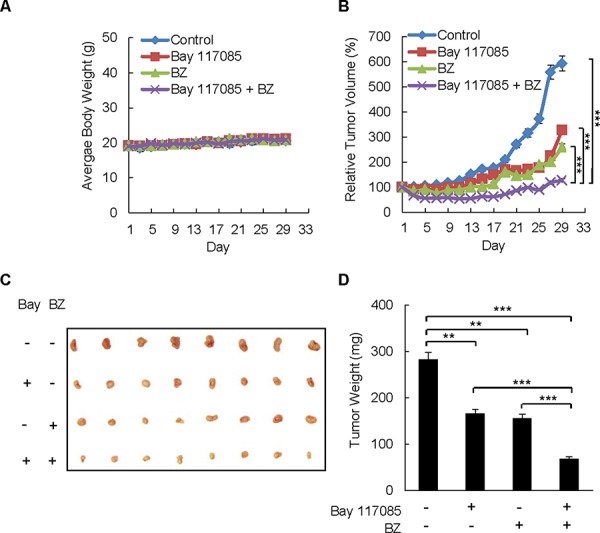
Combination of BZ and Bay 117085 enhances BZ effectiveness in reducing tumor growth in nude mice implanted with ovarian cancer xenografts **A.** Average body weight of mice in four treatment groups (*n* = 8): control, Bay 117085, BZ, and Bay 117085/BZ combination, over the course of four weeks. **B.** Relative tumor volume calculated as the volume at the indicated day divided by the volume at the starting day of treatment. **C.** Images of excised SKOV3 tumors implanted subcutaneously in mice (*n* = 8) after 4 weeks of treatment. **D.** Average weight of the excised tumors (*n* = 8) at the end of the 4-week treatment period. The values in Figure 3 represent the mean +/–SE. Asterisks denote a statistically significant change (**p < 0.01; ***p < 0.001).

To investigate whether Bay 117085 enhances BZ effectiveness in ovarian cancer *in vivo*, we examined the effect of BZ and Bay 117085 alone and in combination on the ovarian tumor growth in nude mice. Female athymic nude mice were implanted (s.c.) with SKOV3 cells. After tumors (~70 mm^3^) developed, the mice were randomly divided into four groups (*n* = 8) injected (i.p.) for 28 days with the following: (a) vehicle control (PBS), (b) Bay 117085 (5 mg/kg) every other day (24), (c) BZ (1 mg/kg) every third day (14), and (d) combination of Bay 117085 (5 mg/kg) and BZ (1 mg/kg). As evaluated by body weight, the treatment regimen was well tolerated since the mice did not lose any weight (Figure 3A).

After 28 days of therapy, Bay 117085 reduced the average relative tumor volume by 40% compared to the control group, whereas BZ reduced the average relative tumor volume by 56% (Figure 3B). Remarkably, the combination of Bay 117085 and BZ significantly decreased the relative tumor volume compared with control animals (79% tumor reduction, *p* < 0.001) or individual treatment with Bay 117085 (61% tumor reduction, *p* < 0.001) or BZ alone (51% tumor reduction, *p* < 0.001) by the final day of treatment. The reduced tumor volume in mice treated with the combination Bay 117085/BZ therapy corresponded to the appearance of the tumors at the end of the treatment (Figure 3C). In addition, the reduced tumor volume in the combination group corresponded to the final average tumor weight at the end of the experiment (Figure 3D). The average final tumor weight in the combination group was reduced by 76% (*p* < 0.001) compared to control mice, 59% compared to Bay 117085-treated mice (*p* < 0.001), and 56% compared to BZ-treated mice (*p* < 0.001) (Figure 3D). These data indicate that combining the IKK inhibitor Bay 117085 and the proteasome inhibitor BZ has significant anti-tumor effectiveness *in vivo*.

### Combination of BZ and Bay 117085 decreases IL-8 expression in nude mice

Since IKK inhibition by Bay 117085 reduced the BZ-induced IL-8 expression in ovarian cancer cells *in vitro* (Figure 2), and since BZ/Bay 117085 combination therapy resulted in the slowest tumor growth *in vivo* (Figure 3), we reasoned that the combination BZ/Bay 117085 therapy might be associated with the reduced IL-8 expression in implanted ovarian cancer xenografts. To analyze the IL-8 expression *in vivo*, we measured IL-8 mRNA levels in tumor samples, and IL-8 cytokine release in plasma samples obtained from mice at the end of the treatment, by using quantitative RT-PCR and ELISA, respectively. Since mice do not have a homolog of the CXCL8/IL-8 gene, which is present in other species including humans [34], the IL-8 detected in mice tumors and plasma samples was derived from the implanted SKOV3 cell xenografts.

**Figure 4:**
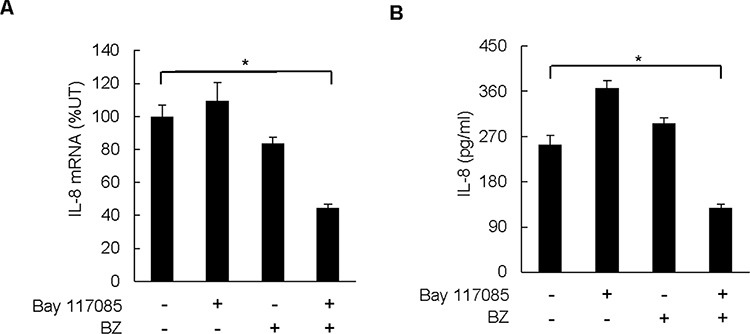
Combination of BZ and Bay 117085 decreases IL-8 expression in nude mice **A.** IL-8 mRNA levels analyzed by real time RT-PCR in excised tumors from the four treatment groups (*n* = 8). **B.** IL-8 cytokine release measured by ELISA in mice plasma samples of the four treatment groups (*n* = 8) at the end of the experiment. The values in Figure 4 represent the mean +/–SE. Asterisks denote a statistically significant change (*p < 0.05) compared to mice in the control untreated group.

Interestingly, while Bay 117085 or BZ alone did not have a significant effect on the IL-8 mRNA levels in tumors, combination of Bay 117085 and BZ significantly decreased the IL-8 mRNA levels in ovarian tumor xenografts (Figure 4A). The IL-8 mRNA levels analyzed in the tumor tissues in the combination group were only 44% compared to the control untreated group (Figure 4A). Correspondingly, the Bay 117085/BZ combination therapy significantly reduced the IL-8 plasma levels, while either drug alone did not inhibit the IL-8 release (Figure 4B). Compared to mice in the control group, the IL-8 plasma levels in the BZ/Bay 117085 combination group were 50% lower (Figure 4B).

### Combination of BZ and Bay 117085 decreases recruitment of S536-p65 to IL-8 promoter in ovarian cancer xenografts

*In vitro*, the IL-8 transcription is regulated by IκBα-independent, promoter specific recruitment of p65 NFκB phosphorylated by IKK at S536 [27, 35, 36]. Thus, we wanted to determine whether the reduced IL-8 expression and tumor growth in the combination group are associated with decreased levels of S536-phosphorylated p65 and its recruitment to the endogenous IL-8 promoter in ovarian cancer xenografts. To evaluate the S536P-p65 levels in tumors tissues, we analyzed p65, S536P-p65, p50, IκBα, and actin protein levels in whole cell extracts prepared from tumor tissues by immunoblotting.

**Figure 5:**
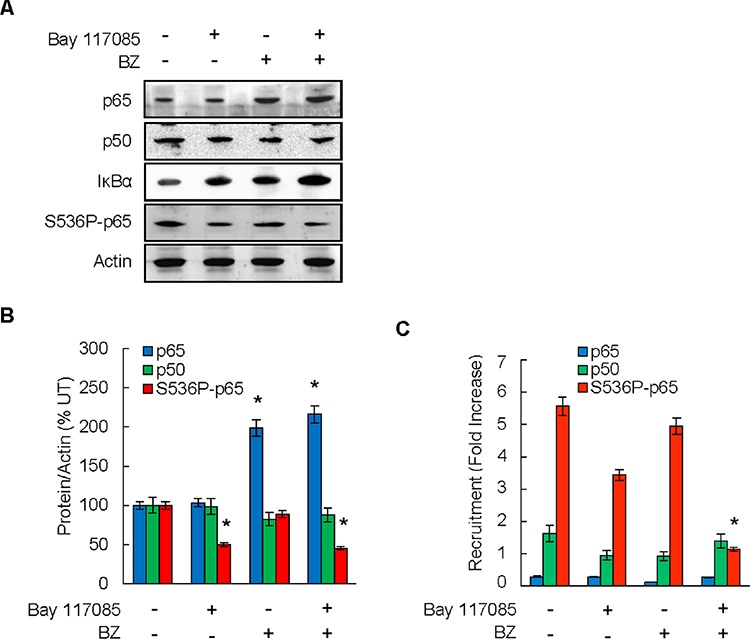
Combination of BZ and Bay 117085 decreases recruitment of S536-p65 to IL-8 promoter in ovarian cancer xenografts **A.** Immunoblotting analysis of p65, p50, IκBα, S536-p65, and control actin in whole cell extracts prepared from excised tumors at the end of the experiment. Representative samples from each treatment group (*n* = 8) are shown. **B.** Densitometric evaluation of p65, p50, and S536P-p65 in whole cell extracts analyzed by immunoblotting in panel A. The values for Bay 117085, BZ, and Bay 117085/BZ were compared to the values for control untreated group, which were considered 100%. The values represent the mean +/−SE (*n* = 8); asterisks denote a statistically significant (*p* < 0.05) change compared to control untreated group. **C.** Recruitment of p65, p50, and S536P-p65 to endogenous IL-8 promoter in implanted tumors in the four treatment groups of mice (*n* = 8) was analyzed by ChIP and quantified by real time PCR. The data are presented as the difference in occupancy of each protein between the IL-8 promoter and the IGX1A (SA Biosciences) negative control locus, and represent the mean +/–SE (*n* = 8). The asterisk denotes a statistically significant (*p* < 0.05) change compared to the control untreated group.

As shown in Figures 5A and 5B, proteasome inhibition by BZ increased the protein levels of p65 in tumor tissues. This is consistent with previous studies demonstrating that p65 undergoes proteasomal degradation [37–39]. In contrast to p65, p50 levels were not substantially changed between the four groups, indicating that p50 is not subjected to proteasomal degradation *in vivo*. Both Bay 117085 and BZ increased the tumor levels of IκBα, which was highest in the combination group; this is consistent with the well-documented IKK-dependent proteasomal degradation of IκBα [40, 41]. The levels of S536-p65 were highest in the control group, and as expected, S536 p65 phosphorylation was significantly reduced in mice treated with the IKK inhibitor Bay 117085 (Figures 5A, 5B).

To determine whether the decreased expression of IL-8 in the combination group is associated with the decreased promoter occupancy of S536P-p65 in tumor tissues, we analyzed S536P-p65 recruitment to the endogenous IL-8 promoter by chromatin immunoprecipitation (ChIP). To this end, tumor tissues were homogenized, proteins and DNA were cross-linked with formaldehyde, and chromatin was sheared by sonication. The recruitment of p65, S536P-p65, and p50 to IL-8 promoter in tumor xenografts was measured by ChIP and quantified by real time PCR. As shown in Figure 5C, p65 and p50 were not substantially recruited to the IL-8 promoter, and their promoter occupancy was not significantly affected by Bay 117085 or BZ. In contrast, S536P-p65 was considerably recruited to the IL-8 promoter in tumor tissues, and its recruitment was significantly decreased in the combination group.

## DISCUSSION

The key finding of this study is that combination of BZ and the IKK inhibitor Bay 117085 significantly reduces tumor growth of ovarian cancer xenografts in nude mice when compared to either drug alone. After four weeks of therapy, the combination of Bay 117085 and BZ inhibited the average tumor volume by 61% compared with Bay 117085 alone, and by 51% compared with BZ alone. The average tumor weight in the combination group was decreased by 59% compared to Bay 117085 alone, and by 56% compared to BZ alone. The reduced tumor growth in mice treated with the BZ/Bay 117085 combination was associated with decreased tumor IL-8 mRNA levels and decreased IL-8 concentration in plasma samples. Furthermore, the reduced tumor growth in the combination group was associated with decreased tumor levels of S536P-p65 NFκB, and with the decreased recruitment of S536P-p65 to IL-8 promoter in tumor tissues. These data provide the first *in vivo* evidence that combining BZ with IKK inhibitor is effective and may demonstrate a clinical benefit in the ovarian cancer treatment.

Despite the limited effectiveness of BZ as a single agent in the treatment of ovarian carcinoma and other solid tumors, BZ has been considered in combination with cisplatin and other platinum drugs [16–18], since it prevents the proteasomal degradation of cisplatin influx transporter, resulting in increased cisplatin uptake and tumor cell killing [19, 20]. The original rationale behind BZ development and use in multiple myeloma was the inhibition of NFκB-dependent transcription of anti-apoptotic genes, by blocking the inducible proteasomal degradation of IκBα and nuclear translocation of NFκB subunits [21, 22]. However, later studies demonstrated that in unstimulated cells, proteasome inhibition actually induces NFκB activity by increasing stability and activity of IKK [27, 42]. Even though in ovarian cancer cells, proteasome inhibition reduces expression of NFκB-dependent anti-apoptotic genes and induces apoptosis [29, 30], it also induces IKK-dependent IL-8 expression [27]. Since suppression of the BZ-induced IL-8 expression increases the BZ pro-apoptotic effect in ovarian cancer cells, and since IL-8 levels correlate with ovarian cancer progression [9–12], these data suggest that the BZ-induced IL-8 expression might represent one of the mechanisms responsible for the decreased effectiveness of BZ in ovarian cancer treatment.

We have recently shown that the mechanisms that regulate the IL-8 expression in ovarian cancer cells *in vitro*, involve IKKβ-dependent S536 phosphorylation of p65, which is specifically recruited to the IL-8 promoter, resulting in increased IL-8 transcription [27]. Interestingly however, while BZ substantially increases the IL-8 expression in ovarian cancer cells *in vitro* [27], BZ alone has only an insignificant effect on the IL-8 mRNA tumor levels and IL-8 plasma concentrations in nude mice. Furthermore, in contrast to inhibiting the BZ-induced IL-8 expression *in vitro*, Bay 117085 alone somewhat increases the IL-8 mRNA tumor levels and IL-8 plasma concentrations *in vivo*. Even though the precise mechanisms are unclear at present, they likely involve additional side effects of BZ and Bay 117085 on other signaling molecules and pathways. In this context, previous *in vitro* studies have shown that in addition to inhibiting the IKK activity, Bay 117085 also has an NFκB-independent effect, and activates mitogen-activated protein kinases (MAPK), extracellular signal-regulated kinases ERK1/2 and p38 kinase in human chondrocytes and monocytic cells, respectively [43, 44]. Since *in vitro* studies have shown that the IL-8 expression in ovarian cancer cells is regulated by p38 and ERK1/2 kinases [45–47], it seems plausible that Bay 117085, as a single agent, increases the IL-8 expression in mice by activating p38 and/or ERK1/2. Intriguingly, however, combination of BZ with Bay 117085 has a synergistic effect on the IL-8 expression in mice, and results in a significantly reduced ovarian tumor growth compared to either drug alone. Even though the regulation of IL-8 expression *in vivo* is complex, and more studies are warranted, one possible mechanism may consist of BZ inhibition of the Bay 117085-induced p38 and/or ERK1/2 pathways, since recent studies have indicated that proteasome inhibition by BZ suppresses MAPK signaling [48, 49]. In this model, an individual treatment with BZ or with Bay 117085 has only a modest effect on reducing ovarian tumor growth in mice since it does not significantly inhibit the IL-8 expression. However, combination of BZ and Bay 117085 maximizes the potential of both drugs to inhibit the IL-8 expression and reduce ovarian tumor growth in *vivo*. In ovarian cancer cells *in vitro*, the IL-8 expression is regulated by IKKβ-mediated recruitment of S536P-p65 NFκB to the IL-8 promoter [27]. Since the decreased expression of IL-8 in mice treated with the BZ/Bay 117085 combination is associated with the reduced S536 phosphorylation of p65 and its decreased recruitment to the IL-8 promoter in tumor tissues, these results suggest that the IL-8 expression *in vivo* is also regulated by S536P-p65.

High expression of IL-8 and IKKβ in ovarian cancer tissues has been related to the aggressive nature of this disease, and to the poor outcome [9–12, 50–52]. Interestingly, epidemiological studies have indicated that the regular use of non-steroidal anti-inflammatory drugs (NSAIDs), including aspirin and diclofenac, is associated with a reduced risk of ovarian cancer [53–56]. In addition, recent studies have shown that NSAIDs reduce ovarian tumor growth in mice [57, 58]. Even though one of the main mechanisms of action of NSAIDs is the inhibition of cyclooxygenase activity, they also inhibit the IKK activity, particularly the activity of IKKβ [59, 60]. Although as single agents, IKK inhibitors have failed to exhibit a strong anticancer effect in ovarian cancer, they have been considered in combination with chemotherapy, particularly with cisplatin and paclitaxel, since they inhibit the cisplatin- and paclitaxel-induced NFκB activity in ovarian cancer cells [31, 61, 62].

Collectively, our data demonstrate that combining the proteasome inhibitor BZ with the IKK inhibitor Bay 117085 significantly reduces ovarian tumor growth in nude mice when compared to either drug alone. Even though additional mechanisms are likely to be involved, our results indicate that the reduced tumor growth is associated with the IKK-dependent decreased S536 phosphorylation of p65 and its decreased recruitment to IL-8 promoter, resulting in reduced IL-8 transcription. To our knowledge, this is the first report demonstrating that IKK inhibition increases BZ effectiveness in reducing ovarian tumor growth *in vivo*, and suggesting that future studies and clinical trials should examine the effect of IKK inhibitors on increasing the BZ effectiveness in ovarian cancer treatment.

## MATERIALS AND METHODS

### Antibodies and reagents

Purified polyclonal antibodies against human p65 NFκB (sc-372), phosphorylated p65 NFκB at S536 (sc-33020), p50 NFκB (sc-7178), and IκBα (sc-371) were purchased from Santa Cruz Biotechnology (Santa Cruz, CA, USA). Purified polyclonal antibody against actin was from Sigma (St. Louis, MO, USA). Horseradish peroxidase (HRP)-conjugated anti-rabbit, anti-mouse and anti-goat secondary antibodies were from Santa Cruz Biotechnology (Santa Cruz, CA). Bortezomib was obtained from Selleck Chemicals (Houston, TX, USA). The IKK inhibitor Bay 117085 was purchased from Cayman Chemicals (Ann Arbor, MI, USA). All other reagents were molecular biology grade and were from Sigma (St. Louis, MO, USA).

### Cell culture

Human ovarian cancer OVCAR3 and SKOV3 cells were obtained from American Type Culture Collection (ATCC; Rockville, MD, USA). The cells were cultured in RPMI 1640 medium (Invitrogen, Grand Island, NY, USA) supplemented with 10% heat inactivated fetal bovine serum (FBS; Invitrogen, Grand Island, NY) and antibiotics as described [27]. Prior to treatment, cells were seeded (5 × 10^5^ cells/ml) for 24 hours in 6-well plates and grown at 37°C with 5% CO_2_. For *in vitro* experiments, bortezomib and Bay 117085 were dissolved in DMSO, and an equivalent DMSO volume was used as a solvent control.

### Transfection with siRNA

Human IL-8 (sc-39631) and non-silencing (sc-37007) small interfering RNAs (siRNAs) were obtained from Santa Cruz Biotechnology (Santa Cruz, CA, USA). Prior to transfection, cells were seeded into a 6-well plate and incubated in a humidified 5% CO_2_ atmosphere at 37°C in antibiotic-free RPMI medium supplement with 20% FBS for 24 h to 80% confluence. For each transfection, 80 pmol of either non-silencing siRNA-A control or IL-8 siRNA (Santa Cruz Biotechnology, CA) were used. Cells were transfected for 6 hours in siRNA transfection medium (sc-36868) with siRNA transfection reagent (sc-29528) according to manufacturer's instructions (Santa Cruz Biotechnology, Santa Cruz, CA). After transfection, fresh medium with antibiotics was added, and the cells were grown for 24 hours before BZ treatment.

### Ovarian cancer xenografts

All animal procedures were carried out in accordance with the recommendations in the Guide for the Care and Use of Laboratory Animals of the NIH, and were approved by the Institutional Animal Care and Use Committee of St. John's University. Five-week-old female athymic nude mice (Taconic Farms, NY, USA) were used for tumor xenograft experiments. Mice were maintained on an alternating 12 hours light/dark cycle with *ad libitum* water and rodent chow as described [63, 64]. Mice were subcutaneously (s.c.) injected in the right flanks with 5 × 10^6^ of SKOV3 cells in 200 μl of serum free RPMI media. Once the average tumor size reached about 70 mm^3^, mice were randomly divided into four groups (*n* = 8). The control group received the control vehicle (PBS) injected intra-peritoneally (i.p) every other day for four consecutive weeks. The “IKK inhibitor” group received Bay 117085 (5 mg/kg) prepared in PBS, injected i.p. every other day. The “BZ” group received BZ (1 mg/kg) prepared in PBS, injected i.p. every third day. The “combination” group received Bay 117085 every other day, and BZ every third day. Mice were weighed, and tumor volume was monitored by caliper measurement every other day. Tumor volumes were calculated using the following formula: Volume = length × width^2^ × 0.5.

At the endpoint, mice were anesthetized using isoflurane inhalant gas and approximately 1 ml of blood was collected from each mouse by cardiac puncture. The blood samples were centrifuged at room temperature in heparinized tubes to prepare plasma samples, which were stored at −80°C for future IL-8 analysis. Immediately after the blood collection, mice were euthanized using carbon dioxide and tumors were excised, measured, weighed, and collected. Each tumor was cut into two parts; one part was flash frozen in liquid nitrogen and stored in −80°C for future protein extraction and chromatin immunoprecipitation (ChIP). The other half was stored in RNA*later* solution for total RNA extraction.

### ELISA

IL-8 release was measured in cell culture supernatants and mice plasma samples by commercially available IL-8 ELISA kit (R&D, Minneapolis, MN, USA) as described [27].

### Apoptosis assay

Apoptosis was evaluated using a cell death detection ELISA kit that quantifies the release of nucleosomes into the cytoplasm (Cell Death Detection ELISAPLUS, Roche, Indianapolis, IN, USA) as described [28].

### Real time RT-PCR

Total RNA was isolated by using RNeasy mini-kit (Qiagen, Valencia, CA, USA). The iScript one-step RT-PCR kit with SYBR Green (Bio-Rad, Hercules, CA, USA) was used as a supermix and 20 ng/μl of RNA was used as template on a Bio-Rad MyIQ Single Color Real-Time PCR Detection System (Bio-Rad). The primers used for quantification of IL-8 and actin mRNA were purchased from SA Biosciences (Frederick, MD, USA).

### Western analysis

Tumor tissues were rinsed with PBS containing 1 mM PMSF and 1% (v/v) anti-protease cocktail (P8340, Sigma), and homogenized in a lysis buffer containing 0.5M Tris-HCl (pH 6.8), 50% glycerol, 10% SDS and 1% (w/v) bromophenol blue, using dounce homogenizer. The homogenized tissues were immediately boiled (7 min) and centrifuged (5 min, 5, 000 *g*). The supernatants were collected and stored at −80°C. The denatured proteins were separated on 12% SDS gels, and analyzed by immunoblotting as described [26–28].

### Chromatin immunoprecipitation (ChIP)

Tumor tissues were homogenized in PBS buffer containing 1% (v/v) anti-protease cocktail (P8340, Sigma) and 1 mM PMSF by using dounce homogenizer. Proteins and DNA were cross-linked by formaldehyde, and cells were washed and sonicated as described [26–28]. The lysates were centrifuged (15, 000 *g*, 10 min, 4°C), and the supernatant extracts were diluted with ChIP dilution buffer and pre-cleared with Protein A/G Agarose (Santa Cruz, CA, USA) for 2 hours at 4°C. Immunoprecipitation was performed overnight at 4°C, with p65, S536P-p65, or p50 antibodies. Following immunoprecipitation, the samples were incubated with Protein A/G Agarose (1 h, 4°C), and the immune complexes were collected by centrifugation (150 *g*, 5 min, 4°C), washed, and extracted with 1% SDS–0.1 M NaHCO_3_. After reversing the cross-linking, proteins were digested with proteinase K, and the samples were extracted with phenol/chloroform, followed by precipitation with ethanol. The pellets were re-suspended in nuclease-free water and subjected to real time PCR. Immunoprecipitated DNA was analyzed by real-time PCR (25 μl reaction mixture) using the iQ SYBR Green Supermix and the Bio-Rad MyIQ Single Color Real-Time PCR Detection System (Bio-Rad). Each immunoprecipitation was performed at least three times using different chromatin samples, and the occupancy was calculated by using the human IGX1A negative control primers (SA Biosciences, Frederick, MD, USA), which detect specific genomic ORF-free DNA sequence that does not contain binding site for any known transcription factors. The results were calculated as fold difference in occupancy of the particular protein at the particular locus in comparison with the IGX1A locus.

The IL-8 primers used for real time PCR were 5′-GGGCCATCAGTTGCAAATC-3′(forward), and 5′-GCTTGTGTGCTCTGCTGTCTC-3′(reverse).

### Statistical analysis

The results represent at least three independent experiments. Numerical results are presented as means ± SE. Data were analyzed by using an InStat software package (GraphPAD, San Diego, CA, USA). Statistical significance was evaluated by using Mann-Whitney U test with Bonferroni correction for multiple comparisons, and *p* < 0.05 was considered significant.

## References

[1] Coleman RL, Monk BJ, Sood AK, Herzog TJ (2013). Latest research and treatment of advanced-stage epithelial ovarian cancer. Nat Rev Clin Oncol.

[2] Nick AM, Coleman RL, Ramirez PT, Sood AK (2015). A framework for a personalized surgical approach to ovarian cancer. Nat Rev Clin Oncol.

[3] Bast RC, Hennessy B, Mills GB (2009). The biology of ovarian cancer: new opportunities for translation. Nat Rev Cancer.

[4] Lengyel E (2010). Ovarian cancer development and metastasis. Am J Pathol.

[5] Vaughan S, Coward JI, Bast RC, Berchuck A, Berek JS, Brenton JD, Coukos G, Crum CC, Drapkin R, Etemadmoghadam D, Friedlander M, Gabra H, Kaye SB (2011). Rethinking ovarian cancer: recommendations for improving outcomes. Nat Rev Cancer.

[6] Waugh DJ, Wilson C (2008). The interleukin-8 pathway in cancer. Clin Cancer Res.

[7] Sarvaiya PJ, Guo D, Ulasov I, Gabikian P, Lesniak MS (2013). Chemokines in tumor progression and metastasis. Oncotarget.

[8] Singha B, Gatla HR, Vancurova I (2015). Transcriptional regulation of chemokine expression in ovarian cancer. Biomolecules.

[9] Xu L, Fidler IJ (2000). Interleukin 8: an autocrine growth factor for human ovarian cancer. Oncol Res.

[10] Huang S, Robinson JB, Deguzman A, Bucana CD, Fidler IJ (2000). Blockade of NFκB Signaling Inhibits Angiogenesis and Tumorigenicity of Human Ovarian Cancer Cells by Suppressing Expression of VEGF and IL-8. Cancer Res.

[11] Merritt WM, Lin YG, Spannuth WA, Fletcher MS, Kamat AA, Han LY, Landen CN, Jennings N, De Geest K, Langley RR, Villares G, Sanguino A, Lutgendorf SK (2008). Effect of interleukin-8 gene silencing with liposome-encapsulated small interfering RNA on ovarian cancer cell growth. J Natl Cancer Inst.

[12] Wang Y, Xu RC, Zhang XL, Niu XL, Qu Y, Li LZ, Meng XY (2012). Interleukin-8 secretion by ovarian cancer cells increases anchorage-independent growth, proliferation, angiogenic potential, adhesion and invasion. Cytokine.

[13] Richardson PG, Mitsiades C, Hideshima T, Anderson KC (2006). Bortezomib: proteasome inhibition as an effective anticancer therapy. Annu Rev Med.

[14] McConkey DJ, Zhu K (2008). Mechanisms of proteasome inhibitor action and resistance in cancer. Drug Resist Update.

[15] Kuhn DJ, Orlowski RZ (2012). The immunoproteasome as a target in hematologic malignancies. Semin Hematol.

[16] Aghajanian C, Dizon DS, Sabbatini P, Raizer JJ, Dupont J, Spriggs DR (2005). Phase I trial of bortezomib and carboplatin in recurrent ovarian or primary peritoneal cancer. J Clin Oncol.

[17] Ramirez PT, Landen CN, Coleman RL, Milam MR, Levenback C, Johnston TA (2008). Phase I trial of the proteasome inhibitor bortezomib in combination with carboplatin in patients with platinum- and taxane-resistant ovarian cancer. Gynecol Oncol.

[18] Aghajanian C, Blessing JA, Darcy KM, Reid G, DeGeest K, Rubin SC, Mannel RS, Rotmensch J, Schilder RJ, Riordan W (2009). Gynecologic Oncology Group. A phase II evaluation of bortezomib in the treatment of recurrent platinum-sensitive ovarian or primary peritoneal cancer: a Gynecologic Oncology Group study. Gynecol Oncol.

[19] Jandial DD, Farshchi-Heydari S, Larson CA, Elliott GI, Wrasidlo WJ, Howell SB (2009). Enhanced delivery of cisplatin to intraperitoneal ovarian carcinomas mediated by the effects of bortezomib on the human copper transporter 1. Clin Cancer Res.

[20] Howell SB, Safaei R, Larson CA, Sailor MJ (2010). Copper transporters and the cellular pharmacology of the platinum-containing cancer drugs. Mol Pharmacol.

[21] Hideshima T, Richardson P, Chauhan D, Palombella VJ, Elliott PJ, Adams J, Anderson KC (2001). The proteasome inhibitor PS-341 inhibits growth, induces apoptosis, and overcomes drug resistance in human multiple myeloma cells. Cancer Res.

[22] Hideshima T, Mitsiades C, Akiyama M, Hayashi T, Chauhan D, Richardson P, Schlossman R, Podar K, Munshi NC, Mitsiades N, Anderson KC (2003). Molecular mechanisms mediating antimyeloma activity of proteasome inhibitor PS-341. Blood.

[23] Vu HY, Juvekar A, Ghosh C, Ramaswami S, Le DH, Vancurova I (2008). Proteasome inhibitors induce apoptosis of prostate cancer cells by inducing nuclear translocation of IκB. Arch Biochem Biophys.

[24] Juvekar A, Manna S, Ramaswami S, Chang TP, Vu HY, Ghosh CC, Celiker MY, Vancurova I (2011). Bortezomib induces nuclear translocation of IκBα resulting in gene-specific suppression of NFκB-dependent transcription and induction of apoptosis in CTCL leukemia. Mol Cancer Res.

[25] Ghosh CC, Ramaswami S, Juvekar A, Vu HY, Galdieri L, Davidson D, Vancurova I (2010). Gene specific repression of proinflammatory cytokines from stimulated human macrophages by nuclear IκBα. J Immunol.

[26] Manna S, Singha B, Phyo SA, Gatla HR, Chang TP, Sanacora S, Ramaswami S, Vancurova I (2013). Proteasome Inhibition by Bortezomib Increases IL-8 Expression in Androgen-Independent Prostate Cancer Cells: The Role of IKKα. J Immunol.

[27] Singha B, Gatla HR, Manna S, Chang TP, Sanacora S, Poltoratsky V, Vancura A, Vancurova I (2014). Proteasome inhibition increases recruitment of IKKβ, S536P-p65 and transcription factor EGR-1 to interleukin-8 (IL-8) promoter, resulting in increased IL-8 production in ovarian cancer cells. J Biol Chem.

[28] Chang TP, Poltoratsky V, Vancurova I (2015). Bortezomib inhibits expression of TGF-β1, IL-10, and CXCR4, resulting in decreased survival and migration of cutaneous T Cell lymphoma cells. J Immunol.

[29] Frankel A, Man S, Elliott P, Adams J, Kerbel RS (2000). Lack of multicellular drug resistance observed in human ovarian and prostate carcinoma treated with the proteasome inhibitor PS-341. Clin Cancer Res.

[30] Bazzaro M, Lee MK, Zoso A, Stirling WL, Santillan A, Shih IeM, Roden RB (2006). Ubiquitin-proteasome system stress sensitizes ovarian cancer to proteasome inhibitor-induced apoptosis. Cancer Res.

[31] Mabuchi S, Ohmichi M, Nishio Y, Hayasaka T, Kimura A, Ohta T, Saito M, Kawagoe J, Takahashi K, Yada-Hashimoto N, Sakata M, Motoyama T, Kurachi H (2004). Inhibition of NFκB increases the efficacy of cisplatin in *in vitro* and *in vivo* ovarian cancer models. J Biol Chem.

[32] Mabuchi S, Ohmichi M, Nishio Y, Hayasaka T, Kimura A, Ohta T, Kawagoe J, Takahashi K, Yada-Hashimoto N, Seino-Noda H, Sakata M, Motoyama T, Kurachi H (2004). Inhibition of inhibitor of NFκB phosphorylation increases the efficacy of paclitaxel in *in vitro* and *in vivo* ovarian cancer models. Clin Cancer Res.

[33] Levêque D, Carvalho MC, Maloisel F (2007). Clinical pharmacokinetics of bortezomib. In vivo.

[34] Nomiyama H, Osada N, Yoshie O (2010). The evolution of mammalian chemokine genes. Cytokine and Growth Factor Rev.

[35] Buss H, Dörrie A, Schmitz ML, Hoffmann E, Resch K, Kracht M (2004). Constitutive and IL-1-inducible phosphorylation of p65 NFkB at serine 536 is mediated by multiple protein kinases including IkB kinase (IKK)-α, IKKβ, IKKε, TRAF family member-associated (TANK)-binding kinase 1, and an unknown kinase and couples p65 to TATA-binding protein-associated factor II31-mediated IL-8 transcription. J Biol Chem.

[36] Sasaki CY, Barberi TJ, Ghosh P, Longo DL (2005). Phosphorylation of RelA/p65 on serine 536 defines an IκB α-independent NFκB pathway. J Biol Chem.

[37] Saccani S, Marazzi I, Beg AA, Natoli G (2004). Degradation of promoter-bound p65/RelA is essential for the prompt termination of the NFκB response. J Exp Med.

[38] Tanaka T, Grusby MJ, Kaisho T (2007). PDLIM2-mediated termination of transcription factor NFκB activation by intranuclear sequestration and degradation of the p65 subunit. Nature Immunol.

[39] Geng H, Wittwer T, Dittrich-Breiholz O, Kracht M, Schmitz ML (2009). Phosphorylation of NFκB p65 at Ser468 controls its COMMD1-dependent ubiquitination and target gene-specific proteasomal elimination. EMBO Rep.

[40] Karin M (1999). How NFκB is activated: the role of the IκB kinase (IKK) complex. Oncogene.

[41] Kanarek N, Ben-Neriah Y (2012). Regulation of NF-κB by ubiquitination and degradation of the IκBs. Immunol Rev.

[42] Hideshima T, Ikeda H, Chauhan D, Okawa Y, Raje N, Podar K, Mitsiades C, Munshi N, Richardson PG, Carrasco RD, Anderson KC (2009). Bortezomib induces canonical NFκB activation in multiple myeloma cells. Blood.

[43] Relic B, Benoit V, Franchimont N, Ribbens C, Kaiser MJ, Gillet P, Merville MP, Bours V, Malaise MG (2004). 15-deoxy-delta12, 14 prostaglandin J2 inhibits Bay 11–7085-induced sustained extracellular signal-regulated kinase phosphorylation and apoptosis in human articular chondrocytes and synovial fibroblasts. J Biol Chem.

[44] Hu X, Janssen WE, Moscinski LC, Bryington M, Dangsupa A, Rezai-Zadeh N, Babbin BA, Zuckerman KS (2001). An IκBα inhibitor causes leukemia cell death through a p38 MAP kinase-dependent, NFκB-independent mechanism. Cancer Res.

[45] Lee LF, Li G, Templeton DJ, Ting JP (1998). Paclitaxel (Taxol)-induced gene expression and cell death are both mediated by the activation of c-Jun NH2-terminal kinase (JNK/SAPK). J Biol Chem.

[46] Xu L, Pathak PS, Fukumura D (2004). Hypoxia-induced activation of p38 mitogen-activated protein kinase and phosphatidylinositol 3′-kinase signaling pathways contributes to expression of IL-8 in human ovarian carcinoma cells. Clin Cancer Res.

[47] Yin J, Yu C, Yang Z, He JL, Chen WJ, Liu HZ, Li WM, Liu HT, Wang YX (2011). Tetramethylpyrazine inhibits migration of SKOV3 human ovarian carcinoma cells and decreases the expression of interleukin-8 via the ERK1/2, p38 and AP-1 signaling pathways. Oncol Rep.

[48] Jazirehi AR, Economou JS (2012). Proteasome inhibition blocks NFκB and ERK1/2 pathways, restores antigen expression and sensitizes resistant human melanoma to TCR-engineered CTLs. Mol Cancer Ther.

[49] Befani CD, Vlachostergios PJ, Hatzidaki E, Patrikidou A, Bonanou S, Simos G, Papandreou CN, Liakos P (2012). Bortezomib represses HIF-1α protein expression and nuclear accumulation by inhibiting both PI3K/Akt/TOR and MAPK pathways in prostate cancer cells. J Mol Med (Berl).

[50] Chen R, Alvero AB, Silasi DA, Kelly MG, Fest S, Visintin I, Leiser A, Schwartz PE, Rutherford T, Mor G (2008). Regulation of IKKβ by miR-199a affects NFκB activity in ovarian cancer cells. Oncogene.

[51] Hernandez L, Hsu SC, Davidson B, Birrer MJ, Kohn EC, Annunziata CM (2010). Activation of NFκB signaling by inhibitor of NFκB kinase beta increases aggressiveness of ovarian cancer. Cancer Res.

[52] Kinose Y, Sawada K, Makino H, Ogura T, Mizuno T, Suzuki N, Fujikawa T, Morii E, Nakamura K, Sawada I, Toda A, Hashimoto K, Isobe A (2015). IKKβ regulates VEGF expression and is a potential therapeutic target for ovarian cancer as an antiangiogenic treatment. Mol Cancer Ther.

[53] Cramer DW, Harlow BL, Titus-Ernstoff L, Bohlke K, Welch WR, Greenberg ER (1998). Over-the-counter analgesics and risk of ovarian cancer. Lancet.

[54] Wernli KJ, Newcomb PA, Hampton JM, Trentham-Dietz A, Egan KM (2008). Inverse association of NSAID use and ovarian cancer in relation to oral contraceptive use and parity. Br J Cancer.

[55] Murphy MA, Trabert B, Yang HP, Park Y, Brinton LA, Hartge P, Sherman ME, Hollenbeck A, Wentzensen N (2012). Non-steroidal anti-inflammatory drug use and ovarian cancer risk: findings from the NIH-AARP Diet and Health Study and systematic review. Cancer Causes Control.

[56] Trabert B, Ness RB, Lo-Ciganic WH, Murphy MA, Goode EL, Poole EM, Brinton LA, Webb PM, Nagle CM, Jordan SJ, Risch HA, Rossing MA, Doherty JA, Australian Ovarian Cancer Study Group, Australian Cancer Study (Ovarian Cancer) (2014). Aspirin, nonaspirin nonsteroidal anti-inflammatory drug, and acetaminophen use and risk of invasive epithelial ovarian cancer: a pooled analysis in the Ovarian Cancer Association Consortium. J Natl Cancer Inst.

[57] Zerbini LF, Tamura RE, Correa RG, Czibere A, Cordeiro J, Bhasin M, Simabuco FM, Wang Y, Gu X, Li L, Sarkar D, Zhou JR, Fisher PB (2011). Combinatorial effect of non-steroidal anti-inflammatory drugs and NFκB inhibitors in ovarian cancer therapy. PLoS One.

[58] Valle BL, D'Souza T, Becker KG, Wood WH, Zhang Y, Wersto RP, Morin PJ (2013). Non-steroidal anti-inflammatory drugs decrease E2F1 expression and inhibit cell growth in ovarian cancer cells. PLoS One.

[59] Yin MJ, Yamamoto Y, Gaynor RB (1998). The anti-inflammatory agents aspirin and salicylate inhibit the activity of IκB kinease-beta. Nature.

[60] Takada Y, Bhardwaj A, Potdar P, Aggarwal BB (2004). Nonsteroidal anti-inflammatory agents differ in their ability to suppress NFκB activation, inhibition of expression of cyclooxygenase-2 and cyclin D1, and abrogation of tumor cell proliferation. Oncogene.

[61] Huang Y, Fan W (2002). IκB kinase activation is involved in regulation of paclitaxel-induced apoptosis in human tumor cell lines. Mol Pharmacol.

[62] Yang YI, Lee KT, Park HJ, Kim TJ, Choi YS, Shih IeM, Choi JH (2012). Tectorigenin sensitizes paclitaxel-resistant human ovarian cancer cells through downregulation of the Akt and NFκB pathway. Carcinogenesis.

[63] Kathawala RJ, Sodani K, Chen K, Patel A, Abuznait AH, Anreddy N, Sun YL, Kaddoumi A, Ashby CR, Chen ZS (2014). Masitinib antagonizes ATP-binding cassette subfamily C member 10-mediated paclitaxel resistance: a preclinical study. Mol Cancer Ther.

[64] Kathawala RJ, Wei L, Anreddy N, Chen K, Patel A, Alqahtani S, Zhang YK, Wang YJ, Sodani K, Kaddoumi A, Ashby CR, Chen ZS (2015). The small molecule tyrosine kinase inhibitor NVP-BHG712 antagonizes ABCC10-mediated paclitaxel resistance: a preclinical and pharmacokinetic study. Oncotarget.

